# Detection of *Brucella abortus* DNA and RNA in different stages of development of the sucking louse *Haematopinus tuberculatus*

**DOI:** 10.1186/1746-6148-9-236

**Published:** 2013-12-01

**Authors:** Gianluca Neglia, Vincenzo Veneziano, Esterina De Carlo, Giorgio Galiero, Giorgia Borriello, Matteo Francillo, Giuseppe Campanile, Luigi Zicarelli, Laura Manna

**Affiliations:** 1 Department of Veterinary Medicine and Animal Production, Federico II University, V. Delpino 1, 80137 Naples, Italy; 2 Istituto Zooprofilattico Sperimentale del Mezzogiorno (IZSM), V. delle Calabrie 27, Salerno, Italy; 3 Istituto Zooprofilattico Sperimentale del Mezzogiorno (IZSM), V. Salute 2, Portici, Naples, Italy

**Keywords:** *Haematopinus tuberculatus*, *Brucella* spp, Zoonosis, Louse, Real time PCR

## Abstract

**Background:**

Brucellosis is considered the world’s most widespread zoonotic infection. It causes abortion and sterility in livestock leading to serious economic losses and has even more serious medical impact in humans, since it can be a trigger to more than 500,000 infections per year worldwide. The aim of this study was to evaluate the role of *Haematopinus tuberculatus*, a louse that can parasitize several ruminants, as a new host of brucellosis. Louse specimens were collected from seropositive and seronegative water buffaloes and divided in 3 developmental stages: adults, nymphs and nits. All samples were separately screened for *Brucella* spp. DNA and RNA detection by Real Time PCR. In particular, primers and probes potentially targeting the 16S rRNA and the *Brucella* Cell Surface 31 kDalton Protein (*bcsp31*) genes were used for Real Time PCR and buffalo β *actin* was used as a housekeeping gene to quantify host DNA in the sample. A known amount of *B. abortus* purified DNA was utilized for standard curve preparation and the target DNA amount was divided by the housekeeping gene amount to obtain a normalized target value. A further molecular characterization was performed for *Brucella* strain typing and genotyping by the Bruce-ladder, AMOS-PCR and MLVA assays. Data were statistically analysed by ANOVA.

**Results:**

*Brucella abortus* DNA and RNA were detected in all developmental stages of the louse, suggesting the presence of viable bacteria. Data obtained by MLVA characterization support this finding, since the strains present in animals and the relative parasites were not always identical, suggesting bacterial replication. Furthermore, the detection of *Brucella* DNA and RNA in nits samples demonstrate, for the first time, a trans-ovarial transmission of the bacterium into the louse.

**Conclusions:**

These findings identified *H. tuberculatus* as a new host of brucellosis. Further studies are needed to establish the role of this louse in the epidemiology of the disease, such as vector or reservoir.

## Background

In recent years an intensification of livestock production systems was observed in many countries, increasing the risk for zoonosis transmission [1]. Among these, brucellosis, an infection caused by bacteria of the genus *Brucella*, represents one of the main zoonosis worldwide. It causes abortion and sterility in livestock leading to serious economic losses [2] and has even more serious medical impact in humans, leading to more than 500,000 infections per year worldwide [3]. Brucellosis has only been controlled and sometimes eradicated in animal reservoirs in developed world by applying strict veterinary hygiene measures, such as control tests, culling infected animals and environment sanitization [3]. Its eradication is even more difficult in developing countries, because of limited resources to indemnify farmers and their emotional attachment to the animals [4]. Furthermore, the existence of mammalian wildlife reservoirs of *Brucella* is an obstacle to brucellosis eradication in some countries [5]. Brucellosis is endemic in most areas of the world due to the difficulties in the application of control/eradication programs and/or other unknown environmental factors may have influenced the spread of the disease.

**Figure 1:**
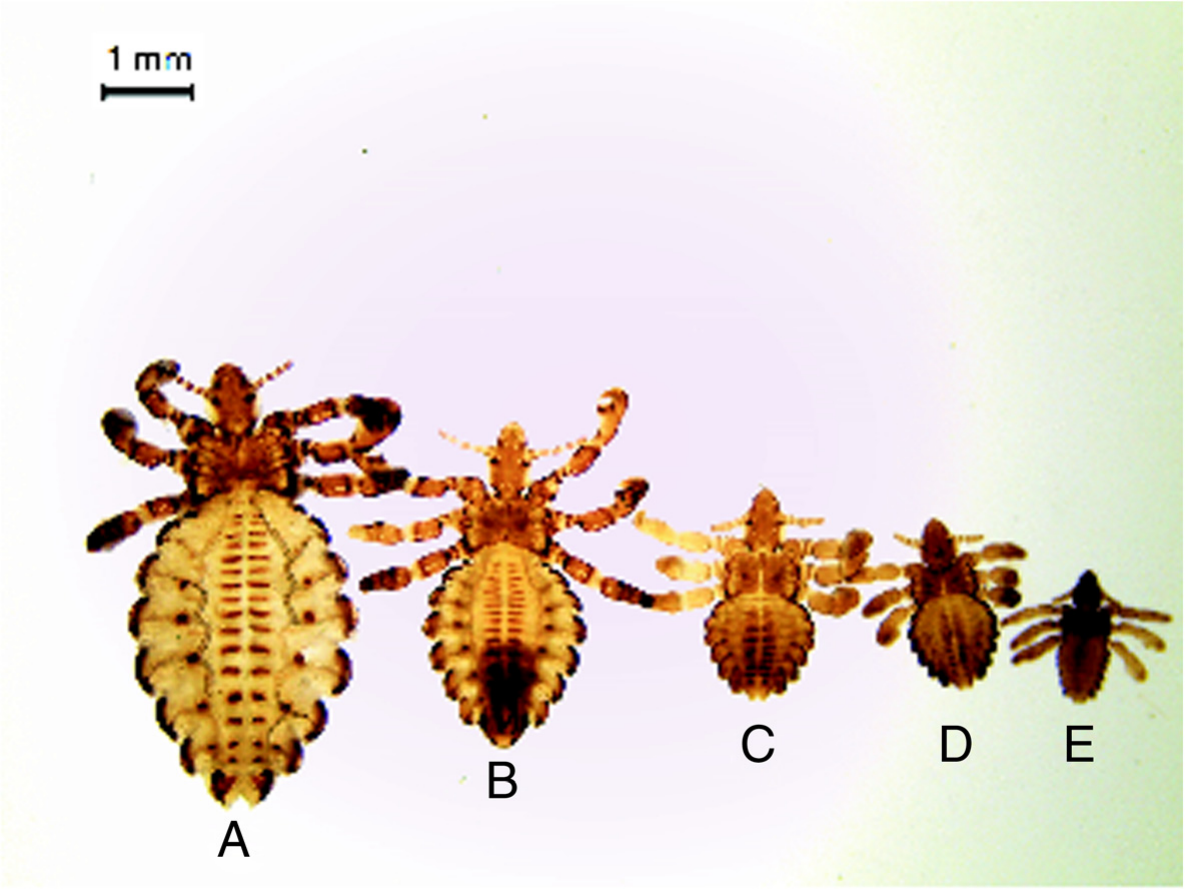
***Haematopinus tuberculatus *** **at different stages: A- female, B- male, C- third stage nymph, D- second stage nymph, E- first stage nymph.**

**Figure 2:**
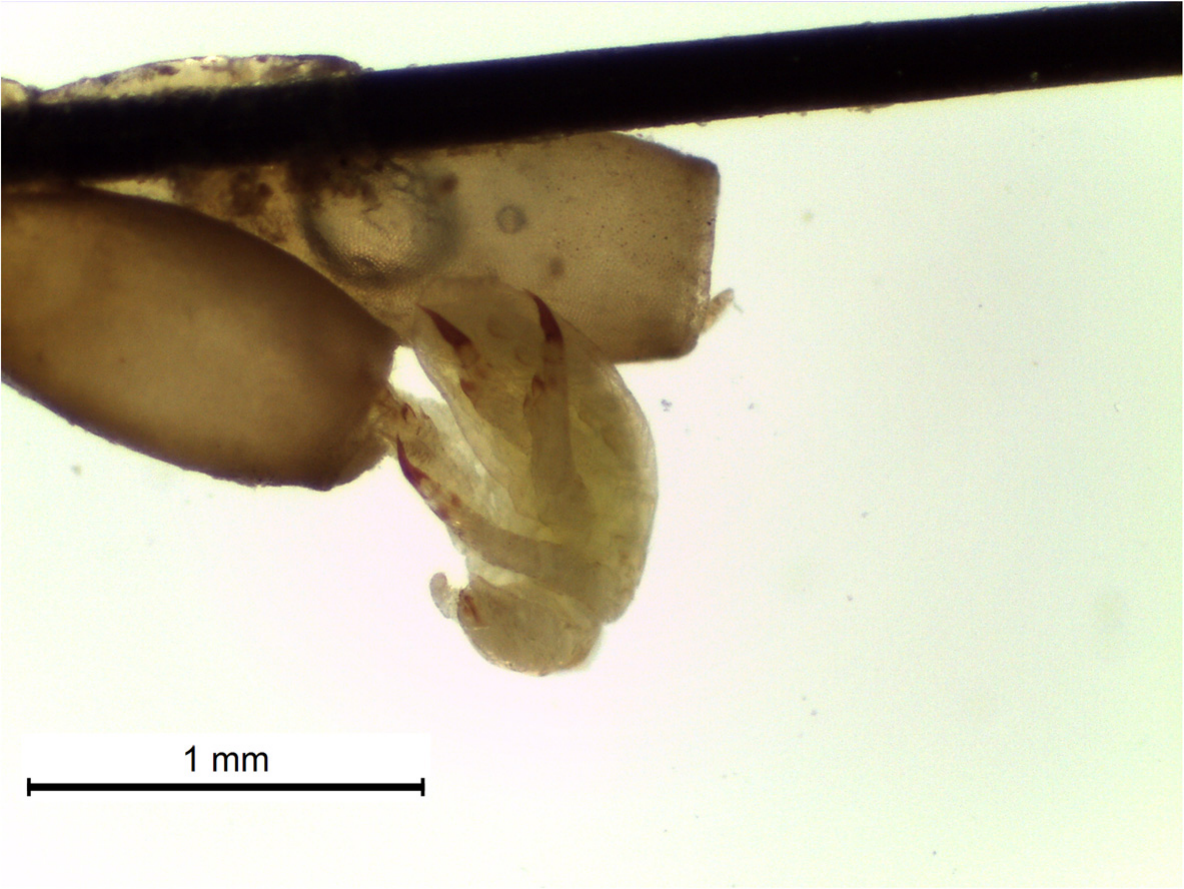
***Haematopinus tuberculatus*: hatching phase of a nit with the emergence of a nymph.**

Recently, great attention has been focused on the role that some insects can play as reservoirs and vectors of many diseases [6,7]. Cheville et al. [8] observed that face flies have only a limited capacity to act as short-term carriers of *B. abortus*, since the bacteria did not replicate in the flies, and some bacteria were found to be degraded by secondary lysosomes of the midgut epithelium. The role of some lice to carry *Brucella* spp. was also hypothesized [9] in Egypt, although the authors failed to detect these bacteria by PCR. Eighteen species of bloodsucking arthropods were identified as natural *Brucella* carriers and 20 species proved to be susceptible to brucellosis infection under experimental conditions [10]. However, no studies have been performed on the sucking louse *Haematopinus tuberculatus* (Figure 1A-1E)*,* Phylum Arthropoda, Class Insecta, Order Phthiraptera, Suborder Anoplura, Family *Haematopinidae*. It has a worldwide distribution, since it has been reported in Asia, Africa, Australia and South America [11]. In Europe it has been described in Albania, Macedonia, France, England and Italy [12-14]. *H. tuberculatus* lives as a permanent ectoparasite and undergoes a simple life cycle. Transition from egg to three nymphal instars to adults (Figure 2), in optimal environmental conditions, is completed on the host in 21–27 days [15]. Cattle [11], camel, bison and water buffalo are susceptible to lice infestation [12,15]. Water buffalo is better adapted to satisfy animal protein demand in tropical countries, where 98% of the world population is bred, but it is also an important milk producer in some developed countries, like Italy, where its breeding has reached a great level of innovation, similar to that in cattle [16]. Recently, it has been demonstrated that *H. tuberculatus* is as potential vector of *Anaplasma marginale*[17]. It may therefore have a similar role in the transmission of other diseases agents, such as *Brucella* spp. In some areas, such as Southern Italy, brucellosis is still endemic despite the application of an eradication program based on a test-and-slaughter approach. Recently, diagnostic molecular techniques have been successfully utilized to identify *Brucella* DNA at genus, species and even biovar levels [18-20]. Real-time PCR constitutes a further technological improvement for the molecular identification and quantification of the genus *Brucella* and for the differentiation of its species. This is a rapid, sensitive and specific diagnostic tool, characterized by a low risk of cross-contamination [21,22]. Furthermore, *Brucella* detection by PCR-based methods is simpler, faster and less hazardous than conventional methods.

The aim of this study was to detect the presence of *Brucella* spp. DNA and RNA in different developmental stages of *H. tuberculatus* in order to evaluate its possible role as vector of this bacterium.

## Methods

### Ethics

The investigation was approved by the Animal Ethics Committee of the University of Naples, Federico II.

### Farms and animals

The animals involved in this study had a naturally-acquired louse infestation. They were bred in a commercial farm located in the South of Italy, where an eradication program based on a test-and-slaughter approach was applied by the Italian Veterinary Health Service. In order to carry out a taxonomic identification, a significant number of lice (about 50) were collected in each farm before the beginning of the trial from 5 randomly selected adult water buffaloes.

Louse specimens were examined on slides under optical (Leica DM 750 HD) and dissection microscopes (Leica EZ4 HD). Species determination was based on morphological keys previously proposed by several authors [11,12,15].

The study was performed on 72 adult water buffaloes bred in six farms located in the South of Italy. Thirty-six infected animals belonging to three farms in which the presence of *Brucella* spp. was detected by the Italian Veterinary Health Service within the National Brucellosis Eradication Program. According to this program, the farms were subjected to periodical controls (every 21 days) from the Italian Veterinary Health Service consisting of conventional serological tests, such as Rose Bengal Test (RBT) and Complement Fixation Test (CFT), on blood samples for the detection of anti-*Brucella* antibodies [23]. The remaining 36 animals originated from three different farms, historically brucellosis-free for at least 20 years, where all the animals were subjected to the same controls every 6 months.

According to RBT and CFT results, 36 seropositive water buffaloes (CFT titre ≥160 I.U.), were selected from the three infected farms (12 water buffaloes for each sampling). Simultaneously, 36 seronegative water buffaloes were randomly selected from the three brucellosis-free farms.

### Louse identification and collection

A total of 6 samples of lice was collected from each animal using an entomological pin and fixed in 70% ethanol. Two samples containing ten adults, two samples containing ten nymphs and two samples containing thirty nits of *H. tuberculatus* were collected in each tube. Three samples (one sample representing each stage) were utilized for DNA extraction, while the remaining were stored at - 80°C for mRNA extraction.

All louse stages were analyzed separately by real-time PCR.

### Blood and tissue collection

Two aliquots of blood samples were collected from the jugular vein of each selected water buffalo. All the samples were taken to the laboratory within two hours of collection. The first was utilized to obtain serum for carrying out RBT and CFT analyses. The second aliquot of whole blood was collected in tubes with EDTA and stored at −20°C until DNA extraction and real-time PCR assay was performed as described below. Seropositive water buffaloes were progressively eliminated according to the Italian brucellosis eradication program, and mammary lymph-nodes were sampled during slaughtering.

### DNA extraction

QIAamp Blood Kit was used to extract DNA from 200 μl of blood samples, mammary lymph-nodes and 100 μl of 10^9^ CFU/ml *Brucella abortus* cultures. DNA extraction from *H. tuberculatus* samples was performed by using the QIAamp DNA Mini Kit (Qiagen, Santa Clarita, CA, USA), according to the supplier’s instructions with some modifications. In particular, the lice were cut, placed in an eppendorf tube, and incubated in the lysis buffer with 50 μl of proteinase K (20 mg/ml) overnight at 56°C. DNA was eluted with 100 μl of the supplied buffer pre-heated at 70°C. The concentration and purity of extracted DNA was assessed by measuring spectrophotometrically the absorbance at 260 nm and 280 nm, respectively, and by gel electhrophoresis.

### Molecular characterization and MLVA analysis

Molecular techniques were carried out on new samples collected from one seropositive farm. In particular, five seropositive water buffaloes were randomly chosen and, from each animal, a new double lice sampling was performed, the first one consisting of the collection of ten adults, ten nymphs and thirty nits of *H. tuberculatus* individually stored; the second one consisting of the collection of ten adults, ten nymphs and thirty nits of *H. tuberculatus* pooled according to the developmental stage in three separate tubes.

*Brucella* strain typing was performed by the Bruce-ladder and AMOS-PCR assays [22] carried out on the DNA extracted from mammary lymph-nodes and parasite pools. The DNA extracted from lymph-nodes and individual parasite samples was analyzed by the MLVA-16 typing technique, as elsewhere described [20,24-26]. The 16 primer pairs were divided into two groups: panel 1 (*loci* Bruce06, Bruce08, Bruce11, Bruce12, Bruce42, Bruce43, Bruce45, and Bruce55) and panel 2 (*loci* Bruce04, Bruce07, Bruce09, Bruce16, Bruce18, Bruce19, Bruce21, and Bruce30). Amplifications were initiated by denaturing the sample for 3 min at 94°C, followed by 30 cycles at 94°C for 30 s, 60°C for 30 s, and 72°C for 50 s. After the last cycle samples were incubated for an additional 7 min at 72°C before they were stored at 4°C. All the forward primers were labeled with a fluorophore (either FAM or Vic or Ned or Pet). PCR products were mixed together in a ratio 1:1:1:1 to obtain four different mixtures each one containing 4 amplicons labeled with 4 different fluorophores. The mixtures were then denatured in presence of Hi-Di formamide and analyzed by capillary electrophoresis with a 310 Genetic Analyzer (Applied Biosystems, Foster City, CA) equipped with a 47 cm long and 50 μm section capillary filled with the separation medium POP-4 polymer. PCR products relative to the *loci* Bruce06, Bruce11 and Bruce42 were also stained with ethidium bromide and resolved by 2.5% agarose gel electrophoresis to visualize eventual amplicons greater than 500 bp.

### RNA isolation and production of cDNAs

Total RNA was extracted from 100 μl of 10^9^*Brucella abortus* cultures and louse samples by using the RNAeasy Mini Kit (Qiagen, Santa Clarita, CA, USA), according to manufacturer’s instructions. The RNA was resuspended in 100 μl of diethyl pyrocarbonate (DEPC) treated water, and stored at −80°C until use.

Synthesis of cDNA was performed by using a reverse transcription system (Im Prom II Reverse Transcription System Promega, Madison, WI, USA).

### Primers and probes

All DNA and cDNA samples were tested by real time PCR by using designed primers and probes potentially targeting the 16S rRNA and the *Brucella* Cell Surface 31kDalton Protein (*bcsp31*) genes, which are highly conserved in 6 species of the genus *Brucella*[27-29]. Buffalo β *actin* was used as a housekeeping gene [30] to quantify host DNA in the sample. All primers and probes were designed by Primer Express Software (Applied Biosystems), according to technical parameters indicating a low level of penalty coupling factor (Table 1). The fluorogenic probes were synthesized by using a FAM reporter molecule attached to the 5′ end, and a TAMRA quencer linked to the 3′ end (Applied Biosystems, Foster City, CA, USA).

**Table 1:** **Original real time PCR primers and probes used in this study for 16S RNA, 31 KDa (bcsp31** ***) *** **and buffalo β actin genes**

	**Forward primer**	**Reverse primer**	**Probe**
16S RNA	5′- GCGCGTAAGGATGCAAACAT -3′	5′- CTTGCCTTTCAGGTCTGC-3′	5′- GGCTCATCCAGCGAAACG -3′
31 KDa	5′- AAACGGTAGGTTGCCTAGAG -3′	5′- AATGCCTTGTAGGTCTTT-3′	5′- TTATCATCCGGTGAAGAC -3′
β actin	5′-CTGGCACCACACCTTCTACAA -3′	5′- GCCTCGGTCAGCAGCA -3′	5′- CCACGCGCAGCTCG -3′

### Real time PCR

Real-time PCR was performed to amplify DNA and cDNA as previously described [31]. Serial 10-fold dilutions of a known amount (2*10^9^ CFU) of *B. abortus* purified DNA were utilized for standard curve preparation. In each real time PCR run, standards, samples, and negative controls were analyzed in triplicate. For each sample, the cycle threshold (Ct) value was calculated by determining the point at which the fluorescence exceeded the threshold limit. The detection range for each set of primers and probe was from 2 × 10^9^ to 2 × 10^1^ CFU. The standard curve, calculated by independent experiments, was linear over an at least 6-log range of DNA or cDNA concentration points, with an average correlation coefficient of 0.988. The difference for each point of the curve was one log factor. The target DNA amount was divided by the housekeeping gene amount to obtain a normalized target value.

### Statistical analysis

Statistical analysis of data was performed by ANOVA [32]. The mean quantity of colony forming unit recorded in samples of *H. tuberculatus* collected from seropositive (n = 36) animals were compared for both 16S rRNA and the *Brucella* spp. Cell Surface 31kDalton Protein (*bcsp31*) genes. The prevalence of positive samples among different groups was evaluated by the chi-square test [32].

## Results

### DNA detection and quantification

The DNA of the *Brucella* spp. 16S rRNA gene was amplified in 55.6% of the adult and nymph samples, collected from seropositive water buffaloes, whereas it was never detected in any sample collected from seronegative animals. In particular, DNA was amplified in both adult and nymph specimens collected from 12 water buffaloes, in adult specimens collected from 12 different water buffaloes, and in nymph specimens from 4 different water buffaloes.

The *Brucella* spp. *bcsp31* gene was amplified in 44.4% of adult and nymph samples collected from seropositive water buffaloes. It was never detected in seronegative water buffaloes. The DNA was amplified in all the adult and nymph specimens collected from 3 water buffaloes, in adult specimens collected from 5 different water buffaloes, and in nymph specimens from 5 different water buffaloes. Interestingly, all the samples positive to *bcsp31* amplification were also positive to 16S rRNA (Table 2).

**Table 2:** **Frequency of detection of *Brucella *** **spp. 16S rRNA and 31 Kd protein genes DNA and cDNA in different samples of *H. tuberculatus *** **collected from seropositive and seronegative buffaloes**

**Sample**	**Frequency (% positive)**							
	**Seropositive buffaloes**				**Seronegative buffaloes**			
	**16S rRNA**		**31 Kd protein**		**16S rRNA**		**31 Kd protein**	
	**DNA**	**cDNA**	**DNA**	**cDNA**	**DNA**	**cDNA**	**DNA**	**cDNA**
**Adults**	24/36 (66.7)^AB^	20/24 (83.3)	8/36 (22.2)	4/8 (50.0)	0/36 (0.0)	NP	0/36 (0.0)	NP
**Nymphs**	16/36 (44.4)^A^	13/16 (81.3)	8/36 (22.2)	4/7* (57.1)	0/36 (0.0)	NP	0/36 (0.0)	NP
**Nits**	16/18 (88.9)^B^	14/16 (87.5)	8/18 (44.4)	5/8 (62.5)	0/36 (0.0)	NP	0/36 (0.0)	NP

The amount of DNA and cDNA were normalized by using the housekeeping (buffalo β actin gene).

*RNA was not detected in one sample.

NP = Not performed.

Values with different superscripts within columns are significantly different (^A,B,^ P < 0.01).

**Figure 3:**
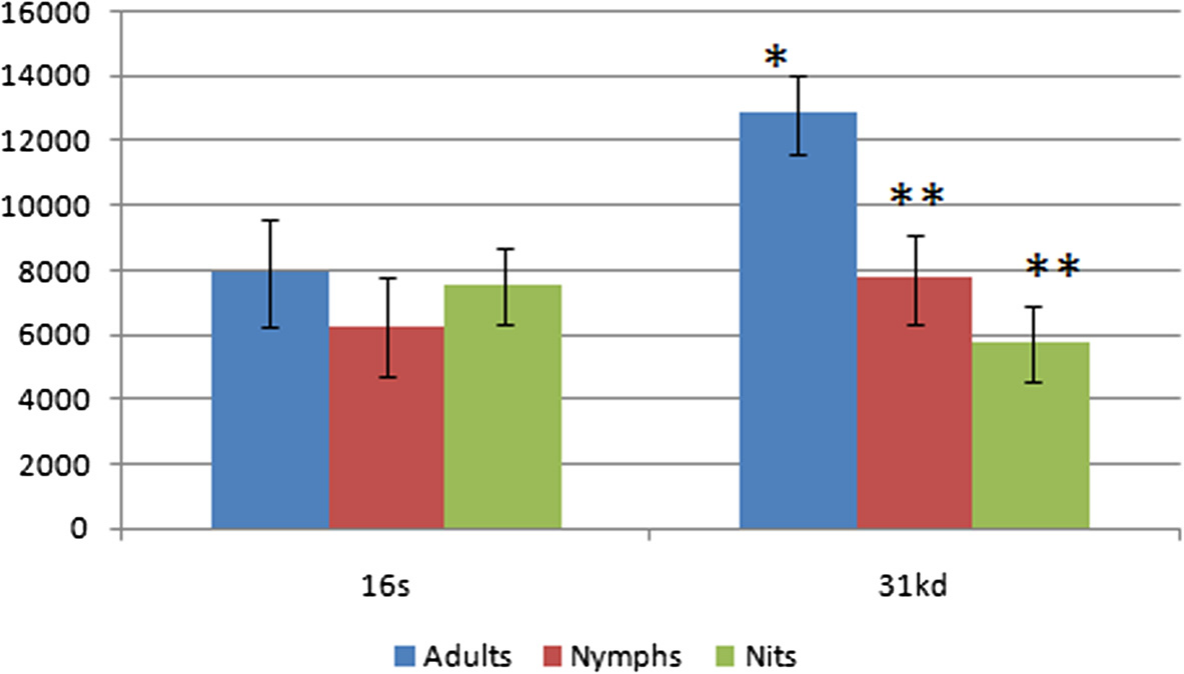
**Mean quantity of *Brucella *spp. DNA amplified in adult, nynphs and nits samples of *Haematopinus tuberculatus *by real time PCR using two different sets of primers and TaqMan probes specific for 16S rRNA and 31 K dalton genes.** Data are expressed as colony forming units per ml. *, **, indicate significant differences (P < 0.05).

Regarding the stage of development (Table 2), the DNA of *Brucella* spp. was amplified in 66.% and 22.2% of the adult specimens, by 16S rRNA and *bcsp31* genes, respectively, whereas it was detected in 44.4% and 22.2% of the nymphs specimens, by 16S rRNA and *bcsp31* genes, respectively. The mean quantity of colony forming units (CFU) per ml amplified by the 16S rRNA gene were similar for both adult and nymph specimens, whereas the CFU amplified by the *bcsp31* gene was higher (P < 0.05) for adults than for nymphs (Figure 3).

Although the nits were collected from all animals, only those belonging to 18 seropositive and 14 seronegative water buffaloes were analyzed by real time PCR assay, since some nits were already hatched, because of the biological cycle of the louse. Interestingly, all samples collected from seronegative animals were negative to real-time PCR, whereas the eggs laid by naturally infected female *H. tuberculatus* lice contained the genomic DNA of *Brucella* spp., as detected by both sets of primers and probes. In this case 88.9% of the samples were positive to 16S rRNA gene detection, whereas the *bcsp31* gene was amplified in only 44.4% of cases (Table 2). In conclusion, the DNA was amplified in at least one stage of louse development in 28 water buffaloes (77.8%).

Real time PCR was unable to detect *Brucella* DNA in any blood samples.

### cDNA detection

As shown in Table 2, bacterial cDNA of 16S rRNA gene was amplified in 83.3, 81.3 and 87.5% of the samples positive to *Brucella* spp. DNA detection in adult, nymph and nit specimens, respectively. Similarly, bacterial *bcsp31* cDNA was amplified in 50.0, 57.1 and 62.5% of the samples positive to DNA detection in adult, nymph and nit specimens, respectively (Table 2).

### Molecular characterization and MLVA analysis

Results from molecular characterization highlighted the presence of *B. abortus* bv. 1 in all lymph-nodes and parasite pools samples. The MLVA assay provided complete genetic profiles from all the lymph-nodes samples and from 38% (19/50) of individual adult parasite samples, 32% (16/50) individual nymph samples and 16% (24/150) individual nit samples. The resulting *Brucella* genetic profiles indicated the prevalence of one main genotype both in water buffaloes and parasites samples (Table 3). Two nymph samples collected from the same water buffalo exhibited 2 different genotypes, and one adult louse sample collected from a different water buffalo showed an additional different genotype (Table 3).

**Table 3:** **MLVA genetic profiles originated from water buffaloes and *H. tuberculatus *** **samples**

**Sample**	**MLVA genotype**
Water buffaloes	4 5 4 12 2 2 3 3 6 21 8 3 7 3 3 4
*H. tuberculatus* adults^*a*^	4 5 4 12 2 2 3 3 6 21 8 3 7 3 3 4
*H. tuberculatus* adult A	4 5 4 12 2 2 3 3 6 21 8 3 **14** 3 3 4
*H. tuberculatus* nymphs	4 5 4 12 2 2 3 3 6 21 8 3 7 3 3 4
*H. tuberculatus* nymph A	4 5 4 12 2 2 3 3 6 21 8 3 7 3 3 **6**
*H. tuberculatus* nymph B	4 5 4 12 2 2 3 3 6 21 8 **11 5** 3 3 **6**
*H. tuberculatus* nits	4 5 4 12 2 2 3 3 6 21 8 3 7 3 3 4

^*a*^All *H. tuberculatus* adult samples except for *H. tuberculatus* adult A.

^*b*^All *H. tuberculatus* nymph samples except for *H. tuberculatus* nymphs A and B; nymphs A and B were collected from the same water buffalo, while the *H. tuberculatus* adult A was collected from a different water buffalo. Underlined and bold data highlight different loci among tested strains.

## Discussion

In this study *Brucella* spp. DNA has been detected in all developmental stages of the sucking louse *H. tuberculatus*. The role of some lice to act as carriers for some diseases has been hypothesized for *Haematopinus eurysternus* and *Haematopinus quadripertusus*[9]. Although the DNA of *Coxiella burnetii* and some species of *Bartonella* were detected by PCR, the authors did not detect *Brucella* spp. DNA [9]. However, in this study lice were randomly collected and it is not specified if the bovines were infected by brucellosis. This aspect may be responsible for the different results recorded in our study.

It is known that both adults and nymphs are hematophagous, hence the blood feeding behavior may explain the presence of *Brucella* spp. DNA. However, two interesting aspects need to be considered. Firstly, real-time PCR assays performed on the blood collected from seropositive animals were negative for *Brucella* spp*.* DNA. This result is in agreement with previous studies [33], in which *Brucella* DNA was detected in milk and lymph tissue samples, rather than in blood. It has been demonstrated that blood is not an adequate substrate to detect the DNA of *Brucella* spp. by real-time PCR, since only a transient, short-lived bacteraemia is described during the infection [23]. Secondly as the bacteria are taken up by macrophages and non-professional phagocytes, only the white cell pellet may be a worthy template for use in PCR detection. In a recent study it was also observed that *Trypanosoma cruzi* is not detected by PCR in the blood of naturally infected wild rodents (*Octodon degus*), while the protozoon is found in the intestinal contents of two species of insect vector (*Triatoma infestans* and *Mepraia spinolai*) [34]. This result was explained by the high rate of parasite amplification of epimastigote forms in the intestines of the insects [34]. It cannot be ruled out that a similar phenomenon occurs for *Brucella* spp. in *H. tuberculatus*. The louse may represent a booster for the bacterium and the target DNA may be present in high copy numbers.

Some studies performed in ticks suggest that the traditional view that arthropods could only acquire infections by feeding on hosts that were parasitaemic, or through transovarial transmission, seems incorrect, since the co-feeding enables microparasite transmission between ticks in absence of a host parasitaemia [35]. Co-feeding alongside infected ticks increases the chances of transmission of microparasites in new-borne ticks, probably increasing also the transmission capability. This phenomenon has been reported for viruses, such as Thogoto virus and TBE group flaviviruses [36], and is probably one of the main routes of transmission for *Borrelia burgdorferi*[37] and *Borrelia afzelii*[38]. In the last case it was demonstrated that a direct passage of spirochetes between co-feeding vector ticks contributes to the likelihood that the Lyme disease spirochete *B. afzelii* perpetuates in nature. Interestingly, a typical scenario in *H. tuberculatus* infestation is the presence of nymphs and adults in clusters (up to 100 specimens in few cm^2^), especially in some specific regions of the animal [12]. This condition may explain the presence of *Brucella* spp. DNA in the lice rather than in blood.

The detection of *Brucella* spp. DNA in nit samples supports an hypothesis of a vertical transmission of bacteria between different phases of development (trans-stadial and trans-ovarial transmissions) of *H. tuberculatus*. Transovarial transmission is considered an important mechanism for maintaining and distributing tick-borne protozoa, bacteria and viruses in nature [36]*: I*n some cases (such as *Rickettsia rickettsii* infection) transovarial transmission, is probably more important in perpetuating infection in nature than the acquisition of the organism from rickettsaemic hosts, as rickettsaemia in mammalian hosts is generally short lived. Since a short-lived bacteraemia is described also during brucellosis, it is likely that engorged *H. tuberculatus* females are able to transfer the bacteria into the nits.

Although a similar CFU mean quantity was recorded in adult, nymph and nit samples, DNA of *Brucella* spp. 16S rRNA gene was detected with different prevalence. In particular, a higher prevalence was recorded in nits (around 90%) compared to adult and nymphs (66.7% and 44.4%, respectively). This may suggest that the rate of infection is relatively high in adult lice, which are able to lay a high rate of infected nits, but probably decreases during the hatching, as observed in *Borrelia* infected ticks [37]. The high resistance of the nits in the environment may also account for *Brucella* spp. survival., especially in some endemic areas.

However, the detection of *Brucella* spp. DNA in lice and nits does not necessarily demonstrate the presence of viable bacteria. As reported above, *Brucella abortus* was isolated also in the flies, but the bacteria were not able to replicate into the carrier [8]. The high stability of DNA molecules and the possibility of its persistence following bacterial death cannot be indicative of the presence of viable microbes. Because of its short half life and lability, RNA has been considered a plausible indicator of viability and a diagnostic target for several microbial infections [39]. The monitoring of bacterial gene expression can be used to characterize the transcriptome of intracellular pathogens and better understand the host:pathogen interaction during infection [40]. Little information is available about *Brucella* spp. gene expression during host:pathogen interaction, because of the difficulty in obtaining an adequate quantity of good quality eukaryotic RNA-free pathogen RNA for downstream applications [41]. The isolation of high-quality bacterial mRNA accurately reflected *Brucella abortus* gene expression and demonstrates the presence of whole and viable bacteria, with replication capability.

This interesting finding is also supported by the MLVA characterization data, since the strains present in animals and the relative parasites were not always identical. This data is indicative of bacterial replication within the parasite and it can not be ruled out that the lice may transmit *B. abortus* infection among the animals, similarly to what has been described for Ixodid ticks in a very old study [42].

## Conclusions

This preliminary study gives a new perspective on the epidemiology of brucellosis and identifies *H. tuberculatus* as a new host of the bacterium. The presence of *B. abortus* DNA and RNA in the nits, confirms the presence of viable and whole bacteria and serves as evidence for bacterial transmission between different developmental stages (trans-stadial and trans-ovarian). Further studies are needed to elucidate the role of *H. tuberculatus* as a possible vector of *Brucella abortus*, by in vitro isolation of the bacterium and experimental infection of animals.

## Competing interests

The authors declare that they have no competing interests.

## Authors’ contributions

GN, VV and LM designed the experiment. VV and MF performed the parasitological investigation. LM and GC designed primer and probes and carried out the real time PCR assay. GG EdC and GB performed the molecular characterization of bacteria by MLVA. GN, GC LZ were involved in drafting the manuscript. LM and VV performed the statistical analysis of the data. All authors contributed to the analysis of the data, discussion of results and implications and commented on the manuscript at all stages. All authors read and approved the final manuscript.

## Authors’ information

GN, VV, and LM are aggregate professors at the department of Veterinary Medicine, Federico II University of Naples.

GC and LZ are full professors at the department of Veterinary Medicine, Federico II University of Naples.

MF is a Veterinary practitioner with long experience in buffalo breeding.

EDC is the Director of Istituto Zooprofilattico Sperimentale del Mezzogiorno (IZSM) – Salerno Section and the head of the National Centre for hygiene, breeding technologies and Buffalo production.

GG is Veterinary Manager at the Istituto Zooprofilattico Sperimentale del Mezzogiorno (IZSM) – Portici Section.

GB is researcher at the Istituto Zooprofilattico Sperimentale del Mezzogiorno (IZSM) – Portici Section.
